# Instrumental In Vivo Assessment of Cosmetic Emulsions Containing Platelet-Rich Fibrin (PRF) or Recombinant Epidermal Growth Factor (EGF): A Pilot Compatibility Study

**DOI:** 10.3390/ph19030394

**Published:** 2026-02-28

**Authors:** Marzena Liliana Wyganowska, Filip Tyliszczak, Marta Marzec, Sylwia Klewin-Steinböck, Izabela Nowak

**Affiliations:** 1 Department of Periodontology and Oral Mucosa Diseases, Poznan University of Medical Sciences, Bukowska 70, 60-812 Poznan, Poland; 2 Private Practice, Krucza 2b, 53-411 Wroclaw, Poland; 3 Department of Applied Chemistry, Adam Mickiewicz University, Poznań, Uniwersytetu Poznańskiego 8, 61-614 Poznan, Poland; marta.marzec@amu.edu.pl (M.M.); nowakiza@amu.edu.pl (I.N.)

**Keywords:** PRF, EGF, skin condition, cream

## Abstract

**Background:** This study evaluates short-term skin compatibility and biophysical changes in new cosmetic preparations containing PRF and EGF, conducted through in vivo studies. **Material and Methods:** The study involved 20 healthy volunteers (aged 20–40) who received three identically packaged creams to be applied for a period of four weeks to specific facial areas: formulation 1: base formulation (control); formulation 2: base formulation human epidermal growth factor (EGF) loaded; and formulation 3: base formulation platelet-rich fibrin (PRF) loaded. Skin assessments were conducted at baseline (week 0) and at weeks 1, 2, and 4. Transepidermal water loss (TEWL), skin hydration using corneometry to determine the moisture content of the stratum corneum, skin elasticity using a cutometer to measure the skin’s ability to return to its original state after deformation, and dermal bioavailability were measured. EGF concentration in the stratum corneum will be measured using the tape-stripping method followed by HPLC (high-performance liquid chromatography) analysis. **Results:** A significant decrease in TEWL was observed for all tested formulations (24%, 37%, and 34%, for formulations 1, 2, and 3, respectively), indicating improved skin barrier function. Formulation 3 showed the highest increase in skin hydration (by 95%), followed by formulation 2. Both formulations 2 and 3 demonstrated improvements in skin elasticity, with formulation 3 showing the greatest enhancement. EGF concentration in the stratum corneum increased over the four-week period, reaching equilibrium with the product concentration by week four. **Conclusions:** The in vivo instrumental compatibility studies confirmed that the new cosmetic formulations were well tolerated and associated with short-term improvement in selected skin parameters.

## 1. Introduction

### 1.1. Epidermis Functions and Repair

The epidermis is the skin’s outer layer, protecting and regulating water and compound transport. It maintains balance in keratinocytes [1,2]. Repair mechanisms include immediate secretion of lamellar bodies from the granular layer, increased synthesis of lamellar bodies, triggered by IL-1α, and enhanced DNA synthesis in epidermal cells due to elevated cytokines [3]. The key signal triggering the release of lamellar bodies is a rapid decrease in Ca^2+^ ion levels, resulting from the loss of these ions, together with water, following disruption of the protective barrier [4]. Synthesis involves enzymes for cholesterol, free fatty acids, and ceramides. Cytokines (IL-1α, IL-1β, IL-6) and growth factors (VEGF, NGF) initiate these processes. TGF-α, EGF, and KGF promote cell proliferation; TGF-β inhibits it [5].

### 1.2. Platelet-Rich Plasma (PRP)

Plasma, the liquid component of blood, is rich in proteins and electrolytes. Various platelet-rich plasma (PRP) preparation protocols have been described in the literature, differing in parameters such as centrifugation steps, relative centrifugal force (RCF), and processing time. These variables directly affect platelet integrity, cellular composition, and ultimately the biological efficacy of the final product. The resulting heterogeneity among PRP preparation systems has led to inconsistencies in terminology and outcomes, posing challenges for standardization and reproducibility across studies. Currently, platelet concentrates are categorized by leukocyte and fibrin content into four types: pure PRP (P-PRP), leukocyte-rich plasma (L-PRP), pure platelet-rich fibrin (P-PRF), and leukocyte-rich platelet-rich fibrin (L-PRF) [6,7].

The use of anticoagulants in many PRP protocols has been shown to interfere with physiological processes such as platelet activation and wound healing, potentially altering the regenerative potential of the preparation [8].

### 1.3. Exploring PRF in Cosmetics

To overcome these limitations, platelet-rich fibrin (PRF) was developed as a second-generation platelet concentrate that requires no anticoagulants or additives and is prepared under lower centrifugal forces. This simplified protocol promotes the formation of a dense fibrin matrix with an optimal distribution of platelets, leukocytes, and growth factors. The resulting product exhibits a slower and more sustained release of cytokines and growth factors, enhancing its regenerative profile. Importantly, injectable PRF (i-PRF) retains sufficient fluidity to be administered in a manner like PRP while offering the additional advantage of fibrin clot formation shortly after injection, thereby providing a natural scaffold within the target tissue.

The composition of PRF can vary based on the equipment and methodology employed, leading to different levels of plasma, erythrocytes, white blood cells, and platelets in the final product.

Fibrin (PRF) stimulates soft tissue healing and repair, with growth factors promoting collagen production and angiogenesis, lasting about two years post-injection [6,9] (Table 1).

Substances penetrate the stratum corneum and dermis transepidermally (through corneocytes and intercellular cement) or via skin appendages. The stratum corneum protects against external factors and prevents water loss, limiting cosmetic substance penetration [10,11]. Most of the studies available in the literature regarding the topical application of PRF concern its use in the treatment of ulcers or burn wounds. Topical application of PRF in skin wound and ulcer management has emerged as a promising adjunctive therapy, particularly in non-healing or deep dermal injuries. Several observational studies and small trials have demonstrated that PRF membranes or gels applied to burn wounds, chronic ulcers, or skin defects accelerate epithelialization and reduce healing time compared to standard care [12]. PRF provides a fibrin scaffold that enables the sustained release of bioactive substances, which enhances fibroblast migration, collagen synthesis, and angiogenesis within the dermal compartment [13,14]. Despite this, most clinical evidence remains limited by small sample sizes, heterogeneity of wound types, and a paucity of large randomized controlled trials in purely dermatologic (cosmetic) contexts.

The aim of the study is the evaluation of the effectiveness of cosmetic preparations containing growth factors (PRF, EGF), which will be conducted through in vivo studies of an application-instrumental nature by determining their impact on selected skin parameters (epidermal hydration level, skin elasticity, transepidermal water loss, skin topography parameters, and macro-texture).

## 2. Results

### 2.1. Physicochemical Properties Study

#### 2.1.1. pH Measurement

The pH analysis of the cosmetic formulations confirmed their appropriate acidity levels, with values ranging from 6.12 ± 0.02 to 7.02 ± 0.02 throughout the 12-week study period. A slight increase in pH was observed with the addition of platelet-rich fibrin (PRF), but it remained within the acceptable range.

#### 2.1.2. Viscosity Measurement

The viscosity measurements indicated that the addition of either the EGF growth factor or PRF slightly increased this parameter. The measured value was in the range of 142,000 ± 500 cP for cream 1, and increased by 1000 ± 500 cP for cream 2 and 2000 ± 500 cP for cream 3.

#### 2.1.3. Stability Analysis

Stability tests using the multi-angle light scattering method revealed minimal changes in the level of backscatter across the sample volume, characteristic of flocculation due to changes in particle size. There were no signs of instability, such as creaming or sedimentation (monitored by the detector), which involves particle migration from the bottom to the top of the sample or vice versa. For formulation 1, TSI values below 1.0 were obtained regardless of the storage time of the formulation. In contrast, for formulations 2 and 3, all TSI values were determined to be similarly low (below 2.5).

#### 2.1.4. Organoleptic Observations

Organoleptic observations, including color and smell, showed that all formulations underwent changes in appearance and odor at higher temperatures (40 °C) due to formulation ingredients decomposition and oxidation. However, all formulations retained their initial smooth, creamy consistency. The only observed changes were in spreadability, which is a natural process and does not indicate instability in the cosmetic formulation; however, it does not arise spontaneously—it is intentionally shaped by the formulation design and the rheological properties of the product.

#### 2.1.5. Microscopic Observations

**Figure 1:**
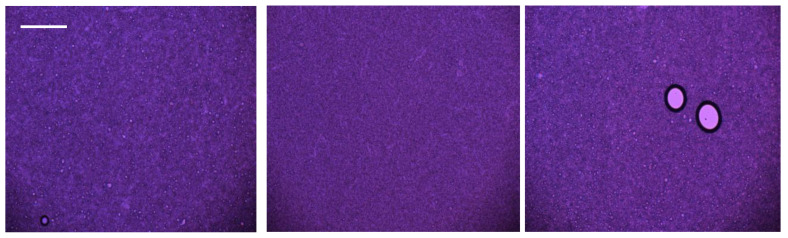
Microscopic photograph of formulations 1–3 (from left to right) at day 84 (samples under the same 40× magnification). Bar = 100 reticle division = 250 µm.

**Figure 2:**
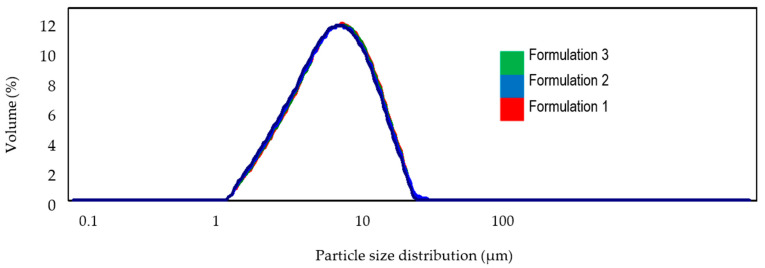
Particle size distribution diagram for 3 formulations after ultrasound treatment.

Microscopic examination demonstrated that all formulations remained stable over the 12-week period, showing no separation within the samples or at their edges. For the formulation containing PRF, the formation of larger particle agglomerates was observed, likely due to the chemical properties of the plasma (Figure 1). There were no inconsistencies in particle size distribution between day 0 and day 84 for any of the formulations (Figure 2).

#### 2.1.6. Summary of Physicochemical Properties

Overall, the physicochemical properties of the cosmetic formulations containing PRF or EGF remained within acceptable ranges, demonstrating good stability, appropriate pH levels, and consistent viscosity over the duration of the study. The minor changes observed did not indicate any significant instability, ensuring that the formulations are suitable for cosmetic use.

### 2.2. In Vivo Studies on a Group of Volunteers

#### 2.2.1. Analysis of Changes in Transepidermal Water Loss (TEWL)

**Figure 3:**
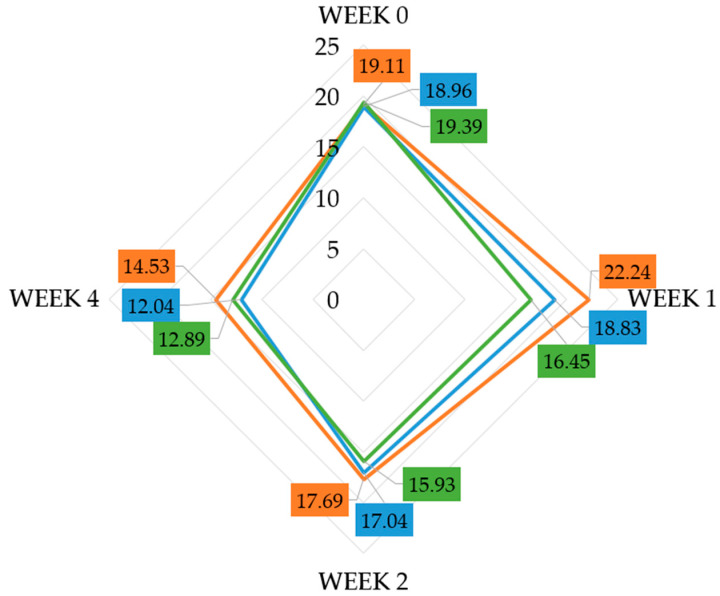
Changes in TEWL values over 4 weeks for all formulations: formulation 1 (orange), formulation 2 (blue), formulation 3 (green).

By analyzing the changes in TEWL values at weeks 0, 1, 2, and 4 across all participants, a favorable average outcome was observed for all tested formulations. The TEWL values for all formulations decreased over 4 weeks. For formulation 1, the TEWL value decreased from 19.11 g/h·m^2^ to 14.53 g/h·m^2^, for formulation 2, from 18.96 g/h·m^2^ to 12.04 g/h·m^2^, and for formulation 3, from 19.39 g/h·m^2^ to 12.89 g/h·m^2^ (Figure 3, Table 2). These results indicate that all formulations were effective in reducing the level of transepidermal water loss in all application sites. However, in the case of the products containing the tested active ingredients (formulations 2 and 3), the effect was more pronounced, with reductions of 36.5% and 33.5%, respectively. Nevertheless, despite these observations, the decrease in TEWL may stem from the occlusive and moisturizing properties of the base formulation (−24.0%), rather than a direct effect of the active ingredients being tested, and the present design does not allow definitive attribution of between-formulation differences independent of facial region. When examining changes after the first week of application, only slight reductions in TEWL were observed for all formulations, and these early improvements were neither substantial nor statistically significant. Post hoc analyses confirmed that statistically significant differences emerged only after two weeks of use (*p* < 0.05). The magnitude of these changes corresponded to a moderate effect size for each formulation—formulation 1 (ε^2^ ≈ 0.12, r ≈ 0.30), formulation 2 (ε^2^ ≈ 0.22, r ≈ 0.42), and formulation 3 (ε^2^ ≈ 0.20, r ≈ 0.40)—indicating that the observed reductions were not only statistically significant but also practically meaningful.

#### 2.2.2. Analysis of Average Moisture Value for Each Cream

**Figure 4:**
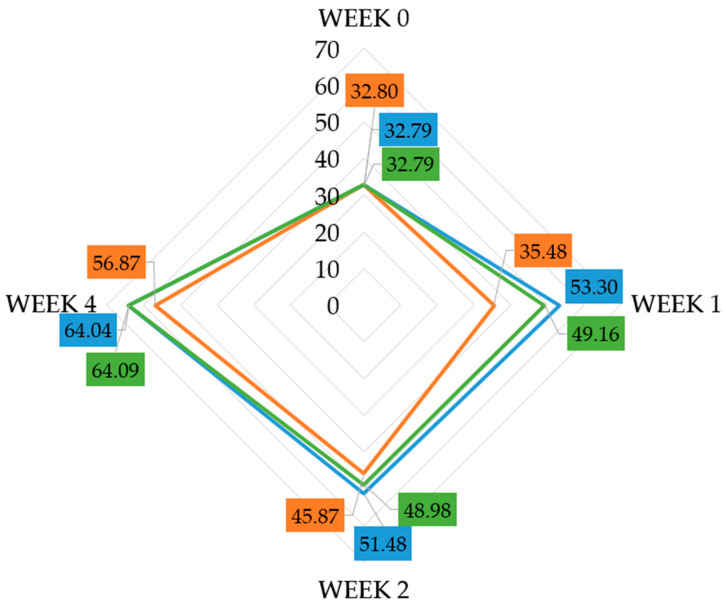
Changes in skin hydration levels over 4 weeks for all formulations: formulation 1 (orange), formulation 2 (blue), formulation 3 (green).

In the analysis of skin hydration levels, a positive impact of the active substances incorporated into the formulations on the skin was observed. The most pronounced improvement occurred with formulation 3, where the hydration value increased from 32.79 CM to 64.09 CM (95.5%). A comparable increase was also noted for formulation 2—95.3% (Figure 4, Table 2). These findings indicate that formulations containing the active ingredients, particularly formulation 3, significantly enhanced skin hydration compared with the base formulation, reaching 73.4%. Post hoc tests revealed that, like the TEWL parameter, statistically significant improvements in hydration were observed for all products only after four weeks of application. The effect sizes suggest a moderate to large practical impact: formulation 1 (ε^2^ ≈ 0.15, r ≈ 0.32), formulation 2 (ε^2^ ≈ 0.23, r ≈ 0.43), and formulation 3 (ε^2^ ≈ 0.26, r ≈ 0.46). Earlier increases noted after the first and second week were detectable but did not reach statistical significance.

#### 2.2.3. Analysis of Average Elasticity Parameter Value

Upon analyzing the selected elasticity parameter R2 (overall skin elasticity, including wrinkling and return to normal state), a favorable outcome was observed for formulations 2 and 3. The elasticity value for formulation 2 increased by five-tenths of a unit (from 0.79 [-] to 0.84 [-]), and for formulation 3, it increased by seven-tenths of a unit (from 0.84 [-] to 0.91 [-]), with a significance level of (*p* < 0.05) (Table 2). The change observed for the base formulation (cream 1) was noticeably smaller (3.8%); nevertheless, it was also considered statistically significant (Table 2). The observed improvements in parameter R2 corresponded to moderate practical effects for each formulation: formulation 1 (ε^2^ ≈ 0.10), formulation 2 (ε^2^ ≈ 0.18), and formulation 3 (ε^2^ ≈ 0.22).

#### 2.2.4. Observation of Skin Topography

**Figure 5:**
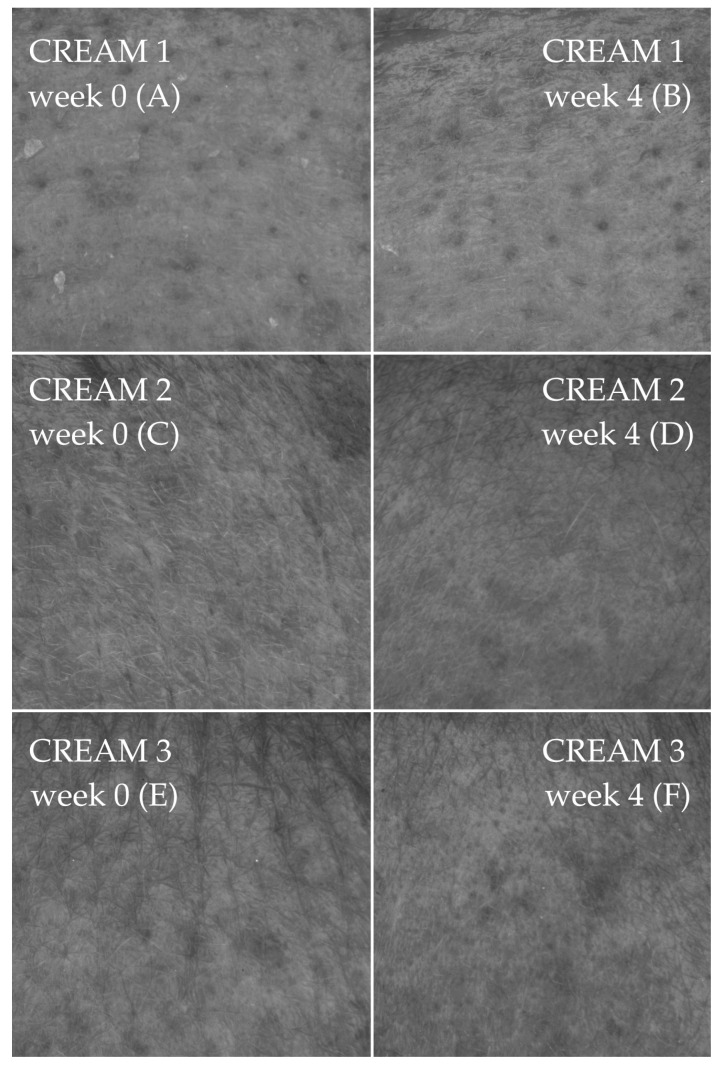
Skin topography images at the application sites of formulation 1 ((**A**) before and (**B**) after 4 weeks), formulation 2 ((**C**) before and (**D**) after 4 weeks), and formulation 3 ((**E**) before and (**F**) after 4 weeks). Image size: 6 mm × 8 mm.

Computer image analysis of the skin allows the surface of the human living skin to be assessed in the form of parameters generally referred to as SELS (Surface Evaluation of Living Skin). As part of the analysis of the microrelief of the skin of the test volunteers, the difference in SELS parameters at weeks 0 and 4 was assessed. The SELS method is based on gray color distribution analysis (obtained using high-resolution photographs) and allows for the calculation of the parameters summarized in Table 3, such as skin roughness (SEr), scaliness (SEsc), and smoothness (SEsm). The analysis of skin topography parameters (SEr, SEsc, and SEsm) revealed distinct differences between the base formulation (cream 1, Figure 5A,B) and the formulations containing active ingredients (creams 2 and 3, Figure 5C–F). For the SEr parameter, all formulations showed a reduction in mean values after four weeks; however, the magnitude of improvement varied considerably. The base cream demonstrated a modest decrease in SEr (−8.9%), whereas creams 2 and 3 showed reductions of −5.3% and −13.6%, respectively. Among these, only cream 3 exhibited a statistically significant improvement (*p* < 0.05; ε^2^ ≈ 0.18), indicating a more pronounced effect associated with the active ingredients. For SEsc (scaliness), minimal change was observed for the base formulation, with a slight reduction of −1.9%, which was not statistically significant. In contrast, cream 2 produced a substantial increase in SEsc (+13.4%), and cream 3 showed a notable reduction of −10.5%. Both changes were statistically significant (*p* < 0.05; ε^2^ ≈ 0.09 and 0.21, respectively), suggesting that the active ingredients in creams 2 and 3 modulated skin scaliness in opposite directions, with cream 3 contributing to a reduction in visible dryness. Regarding SEsm, the base formulation again showed a mild increase in the parameter (+7.6%), but this change did not reach statistical significance. Formulation 2 demonstrated a slight decrease in SEsm (−1.4%), which was also not significant. The most prominent effect was observed with cream 3, where SEsm decreased by −15.9%, a statistically significant improvement (*p* < 0.05; ε^2^ ≈ 0.24), indicating a measurable enhancement in surface smoothness attributable to the active components.

#### 2.2.5. Stratum Corneum Deposition Using the Tape-Stripping Method

Tape-stripping used in this study quantifies analytes present in the stratum corneum only; therefore, the results are interpreted here as stratum corneum deposition and should not be extrapolated to penetration into viable epidermal or dermal layers. EGF values are reported as concentrations measured in the extraction buffer obtained after eluting the collected tape strips (i.e., EGF recovered from removed SC layers), and should not be interpreted as an in situ concentration within viable epidermis/dermis.

In the first week of the study, the concentration of EGF in the stratum corneum ranged from 10 to 15 μg/mL. By the second week, the concentration in the SC for the individual participants ranged from 20 to 25 μg/mL. In the third week, the concentration of the active substance in the stratum corneum increased to a range of 25–30 μg/mL. In the fourth week, the concentration showed a slight further increase. The values for individual participants differed slightly, with the total amount of the active substance not dependent on the depth of tissue penetration, but rather on the thickness of the SC, which varied among the participants.

It should be emphasized that the initial concentration of EGF in the cream was 0.025 mg/g, which is equivalent to 25 μg/g. Therefore, after approximately the fourth week of use, the concentration in the stratum corneum equaled that in the cosmetic product.

## 3. Discussion

To introduce a new cosmetic product into the market, it is obligatory, in addition to conducting shelf life and safety studies, to carry out so-called compatibility studies, preferably in the form of in vivo instrumental tests on human skin.

Platelet-derived cytokines (e.g., PDGF, TGF-β, VEGF, EGF/IGF-1) participate in the renewal processes of the epidermis and dermis during skin regeneration, promoting fibroblast activation, keratinocyte proliferation, angiogenesis, and leading to enhanced extracellular matrix organization [15].

Recent clinical and experimental data indicate that PRF exerts its regenerative effects mainly within the reticular dermis, where dense collagen and elastin bundles determine the skin’s mechanical strength and elasticity. High-frequency ultrasound studies in humans have demonstrated increased dermal density and local thickening after intradermal PRF injections, suggesting remodeling of the deeper dermal layers [16]. It is the thickening that results from increased amounts of elastic and collagen fibers, arranged horizontally [17].

The use of platelet concentrates as additives in topical creams and cosmetic preparations has been only sparsely addressed in the literature. To date, only limited closely related studies are available; thus, the present work expands the evidence by combining a standardized cosmetic formulation approach with short-term in vivo instrumental assessment. At present, only one similar study, but using PRP incorporated into a topical cream formulation, has been described in the literature [18]. The study was conducted on a group of 20 participants of both sexes, aged 30 to 60 years. In contrast to our study, they did not observe any significant differences in TEWL between the two sides of the face. No statistically significant differences between the PRP + serum treatment and the treatment with serum alone were found in the evaluated parameters, like dryness, smoothness, luminosity, radiance, firmness, skin texture, fine facial lines, wrinkles, and hyperpigmentation. An improvement in skin radiance, luminosity, and smoothness was observed in four weeks in both cases. An enhanced effect at 8 weeks was observed with the PRP-enriched serum compared to the serum alone. The differences between the described study and our own study probably could relate to differences in preparing the protocol, which influence the growth factors value and releasing time [11,19]; however, we did not measure the value of growth factors in every sample collected from individuals.

EGF has been used in aesthetics in three emerging categories: in hyperpigmentation, skin restructuring, and facial rejuvenation. In vitro studies have demonstrated that EGF enhances the migration and contractility of aged fibroblasts while simultaneously stimulating hyaluronic acid production and collagen synthesis [20]. These properties highlight EGF’s significant potential as a biological agent for counteracting skin aging and promoting dermal regeneration. Several studies have demonstrated the effectiveness of EGF in the treatment of melasma and in the prevention of post-inflammatory hyperpigmentation after laser treatment through the topical application of EGF-containing serum [21,22].

To date, only one study has investigated the incorporation of EGF into a topical serum. This observational study, conducted on eight patients with atrophic acne scars, reported that all participants experienced visible improvement in scar appearance compared to baseline [23].

EGF is also one of the growth factors detected in PRF. An in vitro study demonstrated that EGF release from PRF reaches its peak approximately 8 h after centrifugation, and then gradually decreases over the following 10 days, as follows: 8 h—250 pg/mL; 24 h—200 pg/mL; 10 days—30 pg/mL [19].

In this study, synthetic EGF was used as a quantifiable marker that can be reliably detected by HPLC after tape-stripping, serving as a proxy for the presence and deposition of growth-factor-related components in the stratum corneum. We acknowledge that PRF contains a complex mixture of cytokines and proteins whose concentrations vary between donors; therefore, EGF quantification should not be interpreted as a direct measure of the overall biological activity of PRF. However, it may serve as an inferred indicator of the overall activity of multiple growth factors present in PRF. The use of synthetic EGF in identical concentrations for all participants enabled a comparative assessment of treatment effects. We did not focus on measuring growth factors in every individual PRF sample, as their release kinetics are highly variable over time. Since the therapy is highly individualized, quantitative comparison of growth factor levels between patients was not meaningful, due to individual differences in plasma composition.

To our knowledge, reports describing the incorporation of platelet-derived preparations or growth-factor-rich fractions into cosmetic emulsions and their evaluation by non-invasive instrumental skin measurements remain limited. Therefore, this work provides application-oriented data on formulation performance and short-term skin parameter changes, complementing the broader PRF/PRP literature.

An important part of the results is the analysis of the impact of the obtained plasma on the properties of the prepared cosmetic formulations. The addition of growth factors or platelet-rich fibrin minimally affected the increase in pH and viscosity of the formulations, with viscosity decreasing over the 12-week period from emulsion preparation. This was particularly noticeable at 40 °C. These results suggest that an emulsion containing growth factors or platelet-rich fibrin may be suitable for use for the specified period if stored at room temperature. These findings were confirmed by freeze/thaw cycle testing.

The viscosity decrease is typically associated with high spreadability, which can be considered a natural physical process occurring in cosmetic formulations; however, it does not arise spontaneously—it is deliberately designed by the formulation design and the rheological properties of the product. The spreadability may occur due to time- and temperature-dependent changes in microstructure and viscosity, and does not necessarily indicate formulation instability. To conclude, the spreadability itself is not a destabilizing factor; however, the detectable changes in spreadability were observed and can affect the epidermal availability [24].

Microscopic examination of samples and backscatter light scattering analysis also confirmed no deterioration in the quality of the emulsions over the tested period [25,26]. According to the assumptions, the application of cosmetic products must not disrupt the acid–base balance within the individual layers of the skin, particularly by compromising the barrier function of the stratum corneum. Therefore, the pH value must fall within the range of 6.0 to 7.5, which was observed for all prepared cosmetic formulations. According to the PAO (Period After Opening) system, the studied time corresponds to the shortest period of usability for commercially used creams.

The second part of the study involved a clinical assessment of skin condition following the application of three emulsions: a base emulsion, one containing growth factor, and one containing platelet-rich fibrinogen. The emulsions were applied twice daily in specific areas for 4 weeks. Weekly measurements were taken of hydration and transepidermal water loss (TEWL), with additional assessments of skin elasticity and topography at the beginning and end of the study.

Proper skin hydration delays visible signs of aging. The dermis contains about 80% water, the epidermis up to 60%, and the stratum corneum approximately 10%. Their condition is crucial for skin appearance, protected by the hydrolipidic film composed of water, lipids, and natural moisturizing factors that retain water in the stratum corneum [2,27]. According to the literature, filaggrin is considered a key factor in binding keratin fibers, ensuring adequate skin hydration. During its degradation, amino acids such as histidine, arginine, and glutamine metabolize into urocanic acid, citrulline, pyroglutamic acid, lactic acid, and urea. The composition of this mixture varies widely depending on individual skin characteristics and seasonal changes. This mixture acts as a humectant, binding water in the outer layers of the epidermis [28,29].

In the conducted study, transepidermal water loss (TEWL) over the course of 4 weeks decreased by an average of 3 units, with specific values for the EGF cream decreasing from 18.96 to 12.04 g/h·m^2^ and, for the PRF cream, a decrease from 19.39 to 12.80 g/h·m^2^ (Table 2). Typical values fall within the range of 8–14, indicating a good functioning hydrolipid barrier. In our volunteers, the mean initial TEWL value was 19.15 g/h·m^2^, which suggests impaired barrier function. The observed decrease in TEWL is consistent with an improvement in barrier integrity. This reduction may be associated with the occlusive and moisturizing properties of the base formulation. It is suggested that the potentially enhanced effectiveness of the EGF-containing cream may be explained by EGF’s stimulatory effect on filaggrin synthesis [30] and epidermal barrier function improvement. This effect may reflect accelerated epidermal renewal and structural reinforcement induced by EGF, with possible secondary normalization on stratum corneum differentiation (including the profilaggrin/filaggrin–NMF (natural moisturizing factor) axis), contributing to stabilized water loss [31]. In the case of PRF, the improvement in TEWL values can be explained by the formation of a semi-occlusive fibrin barrier, which reduces direct water evaporation [32]. High values of the TEWL parameter are an indication of a disruption of the skin’s protective layer (normal function), which is usually associated with a reduced degree of epidermal hydration (but this is not an accurate term). The lower the values of this parameter, the better the condition of the skin and thus the function of the skin barrier [33,34]. What is more, the study results lead to the conclusion that a longer application period (at least two weeks) was required for meaningful changes in skin barrier function to occur.

For both formulations, the achieved hydration was comparable and very good, which was reflected in the skin’s elasticity. It is important to note that the initial measurements differed significantly from those obtained after four weeks and were statistically significant (*p* < 0.05). Comparing the results between the base cosmetic cream (cream 1) and cream 3 containing PRF, the addition of PRF led to a significantly greater increase in skin hydration (by over 20%), accompanied by an improvement in skin barrier function as indicated by a 12% decrease in TEWL. It should be mentioned that the skin required sustained exposure to the formulations to achieve a measurable and reliable enhancement in hydration. This pattern suggests that the active ingredients exert a cumulative effect over time, likely associated with gradual reinforcement of the skin barrier and improved water-binding capacity within the stratum corneum.

The results obtained from the skin topography analysis (SELS) indicate clear differences in the effectiveness of the tested formulations, particularly between the base formulation (cream 1) and the products enriched with active ingredients (creams 2 and 3). In terms of skin roughness (SEr), only cream 3 produced statistically significant improvements (*p* < 0.05), reflected by notable reductions of −13.6%. These findings may suggest that the platelet-rich fibrin enhanced the skin-smoothing effect beyond what could be achieved with the base formulation alone, which showed only a minimal and statistically insignificant change. The effect observed for formulation 3 was particularly pronounced, indicating a superior capacity of its active ingredient to improve the topography of the skin.

A similar pattern was observed for the scaliness parameter (SEsc). While the base cream produced only a marginal reduction (−1.9%), the active formulations elicited divergent responses. After application of Formulation 2, a statistically significant increase in SEsc (+13.4%) was observed. In contrast, cream 3 produced a significant reduction (−10.5%), suggesting an improvement in skin condition through decreased surface flakiness. This divergence highlights that not all active ingredients confer uniform benefits across all aspects of skin topography. For smoothness (SEsm), improvements were again predominantly associated with the active formulations. The base formulation produced a small, non-significant increase in smoothness (+7.6%), whereas cream 2 showed a slight but also non-significant decline (−1.4%). In contrast, cream 3 produced a statistically significant reduction of (−15.9%), indicating a marked improvement in surface uniformity. This result aligns with the observed effects on roughness and scaliness and supports the conclusion that the combination of active substances in cream 3 acts synergistically to enhance overall skin topography. Taken together with the roughness and scaliness data, these results may suggest that the combination of active substances in cream 3 is associated with more favorable changes in skin surface characteristics within the limitations of this short-term assessment.

The limited changes observed for the base formulation confirm that the improvements are unlikely to be attributable merely to the moisturizing or emollient properties of the vehicle, but rather to the specific activity of the added ingredients. Furthermore, the variability in responses between creams 2 and 3 suggests that the type and concentration of active substances may critically influence the direction and magnitude of the skin topography changes. These findings collectively emphasize the importance of selecting optimized active ingredient combinations to achieve targeted improvements in skin surface characteristics. This is likely due to improved skin hydration but may also be the effect of collagen stimulation by growth factors present in the creams [35,36]. According to the literature, growth factors influence keratinocyte proliferation [37] and protect against UV-induced skin damage, which is the primary external factor responsible for aging [38].

Analysis of the measured skin parameters demonstrated that the tested formulations had meaningful effects on skin barrier function, hydration, and elasticity, with the most pronounced benefits observed for formulations containing active ingredients. While some early changes were detectable shortly after the beginning of application, statistically significant improvements typically emerged only after several weeks, particularly for TEWL and skin hydration. These findings underscore the distinction between statistical significance and clinical relevance. Although non-parametric analyses identified significant differences at specific time points, the magnitude of the observed effects suggests that the improvements were not only statistically detectable but also physiologically meaningful (ε^2^ ≈ 0.10–0.26). Reductions in TEWL exceeding 30% for formulations containing active ingredients indicate clear clinical relevance, as such decreases are generally associated with improved skin barrier function. Similarly, the substantial increases in hydration (>90%) for these formulations demonstrate a meaningful enhancement in skin moisture. Improvements in overall elasticity were also practically significant, with formulations containing active ingredients showing the largest gains. Across all parameters, formulations with PRF and EGF consistently outperformed the base formulation, indicating a real functional effect beyond baseline or placebo changes. It is important to note that, due to multiple comparisons in post hoc analyses for some parameters, there is an inherent risk of type I error, which should be considered when interpreting the results. Nevertheless, the consistent pattern of changes across TEWL and hydration supports the practical efficacy of the active formulations (r ≈ 0.40–0.46). Among the assessed SELS parameters, formulation 3 consistently demonstrated statistically significant improvements, with effect sizes indicating moderate to substantial practical impact (ε^2^ ≈ 0.18–0.24). These effect sizes highlight that the changes are not only statistically detectable but also meaningful in terms of skin surface characteristics, complementing the earlier observations for TEWL, hydration, and elasticity. An effect size was also calculated for formulation 2 for the SEsc parameter, yielding ε^2^ ≈ 0.09, slightly below the threshold for a moderate effect, indicating a relatively small practical impact despite statistical significance. The reductions in SEr and SEsc, along with the improvement in SEsm, underscore that the PRF in formulation 3 exerts a real functional effect on the skin topography, beyond the modest effects seen for the base formulation. Importantly, the effect sizes offer a quantitative assessment of clinical relevance, showing that the observed changes are meaningful and extend beyond statistical significance alone.

The tape-stripping results indicate progressive deposition of EGF in the stratum corneum over the 4-week application period. Because this method samples only the outer barrier layers, the findings should not be interpreted as direct evidence of penetration into viable dermal compartments or as proof of growth-factor-driven biological activity in deeper skin layers. In the first week of the study, the concentration of the compound in the stratum corneum ranged from 10 to 15 μg/mL and increased to 25–30 μg/mL by the third week. By the fourth week of the experiment, the concentration did not undergo significant further changes. This examination was possible only for standardized EGF, not for PRF, but EGF is one of the growth factors secreted by platelets. In recent years, a few techniques have been developed for the objective measurement of skin properties, and several commercially available devices have found application in dermatocosmetic research. The dynamics of physiological skin processes are investigated using the non-invasive biophysical measurement tools [33,39]. Nevertheless, the observed changes in biophysical parameters (hydration, TEWL, elasticity, and topography) are consistent with improved skin condition under the study conditions. None of the patients reported any side effects, such as irritation, allergic reaction, itchiness, or pain after application. All of them found all three formulations to be smooth and pleasant to use.

The study was designed as an exploratory pilot study. The limitations are the short study duration and lack of long-term safety or efficacy data. An additional limitation of this study is that only female volunteers were included. Furthermore, because the formulations were applied to different facial regions with distinct baseline biophysical properties, the findings primarily support within-site longitudinal changes over time rather than definitive between-formulation comparisons. The concentrations of EGF and PRF were selected as rational and practical for a proof-of-concept assessment, rather than claiming to be “optimal” in a strict pharmacodynamic sense, and require future work. Concentrations of EGR and PRF were not measured during the study.

## 4. Materials and Methods

### 4.1. Biological Material

The biological material used in this study was platelet-rich fibrin (PRF), collected from 20 female patients aged 20 to 40 during a standard mesotherapy procedure, after obtaining written consent for participation in the study. The study was approved by the Bioethical Committee of the Poznan University of Medical Sciences under resolution number 308/18 of 8 March 2018. The study was registered at ClinicalTrials.gov (NCT07359924) on 21 January 2026. Intact skin at baseline (no active dermatoses, no recent procedures), standardized wash-out (no actives/exfoliants), controlled environment for measurements, and monitoring for irritation/adverse events at each visit were assured. We also clarified that tape-stripping assesses stratum corneum deposition and that integrity was monitored instrumentally (e.g., excluding inflamed sites). Part of the individually collected PRF (from intravenous blood) was used for the mesotherapy treatment, which was performed only on the neck and décolletage areas in every patient. The remaining portion of PRF was used for suspension in an individual cream. Thus, the PRF used in the cosmetic formulation was autologous: PRF was collected from each participant, and the remaining portion (after the mesotherapy procedure) was incorporated into that same participant’s individual cream (no cross-donor use). Exclusion criteria for the study included systemic diseases, smoking, blood disorders, acute viral infections, skin inflammations, pregnancy, and breastfeeding. All participants have II/III skin type according to the Fitzpatrick scale [40].

To prepare the PRF, a sterile RegenLabKit (Crissier, Switzerland) (standardized by Choukroun) set equipped with a 21G butterfly needle and a vacuum blood collection system was used. Blood was drawn into appropriate tubes following the Choukroun method and subjected to centrifugation in a Choukroun centrifuge according to Protocol IV—10 mL of full blood was centrifuged for 3 min (700 rpm, 60× *g*). This protocol employs the low-speed centrifugation concept (LSCC), which allows for the collection of leukocytes, platelets, and growth factors [19].

### 4.2. Syntetized Growth Factor

Human epidermal growth factor (EGF, purity of ≥98%; HPLC grade) with the peptide sequence NSDSECPLSH DGYCLHDGVC MYIEALDKYA CNCVVGYIGE RCQYRDLKWW ELR was used in this study.

### 4.3. Cosmetic Formulations

Ingredients for the oil phase, using Creagel EZ^®^ 7 and Alphaflow^®^ 20 (Créations Couleurs, Dreux Cedex, France), were measured into a glass beaker and thoroughly mixed using a glass rod for 2 min. Subsequently, demineralized water containing a broad-spectrum preservative system Microcare^®^ SB (Thor GmbH, Erfurt, Germany) and the active substance was gradually added to the mixture, with continuous vigorous stirring to form an emulsion until the desired consistency, such as a mayonnaise without any lumps, was achieved, i.e., approximately 3 min (Table 4). The resulting cosmetic formulation was homogenized using a YellowLine DI 25 Basic homogenizer (IKA, Breisgau, Germany, speed rate: 9500 rpm; time: 5 min). Three creams were prepared: the base formulation (formulation 1), the EGF-loaded emulsion, 0.025 mg/g EGF (2.5 mg/100 g cream) (formulation 2), and the platelet-rich fibrin-loaded emulsion, 2 mL/100 g of cream (formulation 3).

### 4.4. Physicochemical Studies

#### 4.4.1. Stability Tests

All cosmetic formulations underwent standard stability tests (ISO/TR 18811:2018(en) [41]), as commonly performed for cosmetic and pharmaceutical products marketed for public use. The emulsions were assessed for their physicochemical properties (viscosity, pH, emulsion type), active substance stability (using HPLC—Table 5 and multiple light scattering, MLS, methods), microbiological purity for approval for testing on volunteers, stratum corneum deposition through stratum corneum stripping tests, and in vivo application tests.

#### 4.4.2. Stability Evaluation

The stability tests of the formulations were carried out immediately after preparation and after 60 days of storage at 40 °C, 75% humidity (climate chamber, KBF260 model, Binder GmbH, Tuttlingen, Germany). The following physical parameters of the tested formulations (3 samples of the same batch) were then examined: spreadability, general appearance (presence of lumps, phase separation, etc.), color (organoleptically), and odor.

#### 4.4.3. pH Measurement

The pH of the cosmetic emulsions was measured three times using the EcoSense^®^ pH 10 pH/Temperature Meter, Pen Style (VMR International, Radnor, PA, USA). The samples were tested as-is. From the obtained results, the arithmetic mean and standard deviation were calculated.

#### 4.4.4. Viscosity Measurement

The viscosity of the cosmetic emulsions was measured three times using the RC02 rotational viscometer (Rheotec, Ottendorf-Okrilla, Germany). The samples were tested without any additional preparation. From the obtained results, the arithmetic mean and standard deviation were calculated.

#### 4.4.5. Microscopy Studies

Each sample was examined under a polarizing microscope (Delta Optical Genetic Pro Trino microscope, 10–40× magnification, Mińsk Mazowiecki, Poland). The samples were tested without any additional preparation after being spread on microscopic glass. For each formulation, images were collected from multiple randomly selected fields of view. InfraView-based analysis enabled rapid screening of the entire field for the presence of micro-aggregates/inhomogeneities.

#### 4.4.6. Light Scattering Stability Analysis

Stability analysis of the formulations was performed using the multiple light scattering method with the Turbiscan Lab Expert device (Formulaction SA, Toulouse, France). The samples were tested as-is.

#### 4.4.7. Stability Testing Points

Stability tests for all formulations included four measurement points, analyzing the samples after 2, 4, 8, and 12 weeks of storage in climate chambers. The comprehensive testing ensured that the formulations maintained their desired properties and remained well-tolerated and associated with beneficial changes.

#### 4.4.8. Growth Factor Quantification in Cosmetic Formulations

The quantification of the growth factor in the obtained cosmetic formulations was conducted using a high-performance liquid chromatograph (HPLC) Varian 920-LC (Agilent Technologies, Santa Clara, CA, USA), equipped with an automatic injector and sample feeder. Both qualitative and quantitative methodologies were developed for the analysis of cosmetic preparations. An amount of 1 g of cosmetic was weighed into a 10 mL polypropylene centrifuge tube and dissolved in 5 mL of a buffer solution of 7.5 mM sodium phosphate (pH 6.8). The solution was mixed for 10 min and centrifuged at 10,000 rpm for 3 min. The eluent was passed through a nylon filter membrane (0.45 μm), and 10 μL filtrate was injected into the HPLC.

#### 4.4.9. Stratum Corneum Deposition Study

The stratum corneum deposition study was performed using the stratum corneumstripping method. This technique involves the sequential removal of the outermost layers of the stratum corneum using Instant d-Squame^®^ Skin Indicators adhesive tape (Dallas, TX, USA) (0.5 in. (12.7 mm) diameter). The removed tape strips (30 for each volunteer, as recommended by Solberg et al. [42]) were then altogether immersed in a buffer solution of 7.5 mM sodium phosphate (pH 6.8) and subjected to ultrasound treatment for 30 min. Following this, the concentration of EGF was determined using the HPLC method (with 85% extraction recovery evaluated using a spike-and-recovery approach). The obtained values represent EGF recovered from removed stratum corneum layers into the extraction buffer and should not be interpreted as evidence of penetration into viable dermis.

### 4.5. Application Study

The application panel consisted of 20 female volunteers. In this research area, pilot studies commonly enroll 15–25 participants, which is considered sufficient for preliminary assessment of safety and biological responses. Volunteers received three uniformly appearing creams, along with instructions for application to specific facial areas, every morning and evening. Cream allocation to facial areas was single-blinded across subjects to prevent site-specific bias—base cream was always on the forehead, and formulations 2 and 3 around the eye area (peri-orbital area). During the study, volunteers were asked not to change any daily routine in diet and hygiene, and to avoid other cosmetic products in the observed areas. Due to the use of a blood-related product, the study was designed only for 4 weeks observation, which can be a limitation. Skin reassessment was conducted after 1, 2, and 4 weeks from the start of application. During the four weeks of the study, baseline measurements (skin hydration and transepidermal water loss) were taken weekly, with elasticity and skin topography additionally examined in weeks 0 and 4. To measure the hydration of skin, an apparatus called a corneometer is used (Corneometer^®^ CM 825, Courage + Khazaka electronic GmbH, Köln, Germany), which allows an accurate, repetitive, and objective measurement of the hydration level of the stratum corneum of the skin. It records changes in electrical capacitance, consistent with the water content of the skin. Another parameter describing the state of our skin is transepidermal water loss (TEWL, Tewameter^®^ TM 300; Courage + Khazaka electronic GmbH, Köln, Germany), and it is one of the most important parameters used to assess the state of the water–lipid layer and thus the barrier function of the skin [33]. Overall skin elasticity was measured using a Cutometer^®^ MPA 580 (Courage + Khazaka electronic GmbH), a device that assesses the mechanical properties of the skin by applying controlled negative pressure and recording the extent and speed of skin deformation. Among the available parameters, only the R2 value was used, as it represents the overall elasticity ratio, providing the most reliable and comprehensive assessment of the skin’s ability to return to its original state after deformation [43]. Parameters related to skin topography were assessed using the Visioscan^®^ VC98 (Courage + Khazaka electronic GmbH). The measurement is based on capturing a high-resolution grayscale image of the skin’s surface, from which the SELS (Surface Evaluation of Living Skin) parameters are subsequently calculated. Three indicators were evaluated: SEr, SEsc, and SEsm. SEr (roughness) reflects irregularities (wrinkles, depressions) on the skin surface (dark pixels in the image). SEsc (scaliness) represents the degree of skin dryness (white pixels), and SEsm (smoothness) indicates the level of surface evenness and fine texture (a narrow histogram of gray levels) [44]. The results collected from the application tests were subjected to statistical evaluation using Statistica 13.3 (StatSoft, Kraków, Poland). Changes in skin parameters were examined for statistical significance with the non-parametric Kruskal–Wallis test. When significant differences were identified, corresponding post hoc procedures were applied (for TEWL and skin hydration). Statistical significance was defined as a *p*-value below 0.05. Effect sizes were calculated for all statistically significant changes, including ε^2^ and r values, starting from approximately 0.10 and 0.30, indicating moderate practical effects. Because formulations were applied to different facial regions with distinct baseline biophysical properties, this pilot study primarily supports within-site longitudinal changes over time.

## 5. Conclusions

In this short-term, single-center pilot study in 20 healthy female volunteers, creams containing the liquid PRF fraction with EGF were well tolerated and were associated with improvements in selected biophysical skin parameters compared with the base and the EGF-only formulation. Tape-stripping/HPLC showed an increasing level of EGF measured in the stratum corneum over four weeks, indicating progressive deposition in the outer barrier layer under the study conditions. An observational trend toward improved skin elasticity and hydration was noted for formulations containing PRF compared with those containing only EGF. Larger, longer, and more diverse studies are required to confirm efficacy, assess long-term safety, and clarify mechanisms.

## Figures and Tables

**Table 1:** Growth factors present in platelet-rich plasma/fibrin and their relevant biological actions [6,9].

Growth Factor	Cosmetic Effects
Platelet-derived growth factors PDGF-αα, ββ, αβ	- Has a chemotactic effect on fibroblasts and macrophages. - Has a mitogenic effect on fibroblasts and endothelial cells.
Transforming growth factor TGF-β1, TGFβ2	- Mediates angiogenesis. - Has a chemotactic effect on fibroblasts, keratinocytes, and macrophages. - Has a mitogenic effect on fibroblasts and smooth muscle cells. - Inhibits endothelial cells, keratinocytes, and lymphocytes. - Regulates matrix proteins, including collagen.
Vascular endothelial growth factor VEGF	- Chemotactic effect on endothelial cells. - Mediates angiogenesis.
Epidermal growth factor EGF	- Mediates angiogenesis. - Has a mitogenic effect on fibroblasts, endothelial cells, and keratinocytes.
Hepatocyte growth factor HGF	- Mediates regeneration.
Fibroblast growth factor FGF	- Mediates in the organization and tissue regeneration.
FGF-9	- Promotes the formation of new hair follicles.

**Table 2:** Comparison of skin biophysical parameters in the study group before and after treatment.

Parameter	Cream No.	Week 0		Week 4		Change [%]	*p*
		Mean	SD	Mean	SD		
TEWL [g/h·m^2^]	1	19.11	0.41	14.53	1.37	−24.0	<0.05
	2	18.96	0.89	12.04	0.70	−36.5	<0.05
	3	19.39	0.35	12.89	0.72	−33.5	<0.05
Skin hydration [CM]	1	32.80	4.71	56.87	5.93	73.4	<0.05
	2	32.79	3.98	64.04	5.30	95.3	<0.05
	3	32.79	5.74	64.09	5.89	95.5	<0.05
Overall elasticity, R2 [-]	1	0.75	0.06	0.78	0.04	3.8	<0.05
	2	0.79	0.04	0.84	0.04	6.7	<0.05
	3	0.84	0.05	0.91	0.04	8.3	<0.05

**Table 3:** Comparison of skin topography parameters in the study group before and after treatment.

Parameter	Cream No.	Week 0		Week 4		Change [%]	*p*
		Mean	SD	Mean	SD		
SEr (roughness)	1	3.85	0.96	3.50	0.73	−8.9	n.s.
	2	3.89	0.32	3.69	0.44	−5.3	n.s.
	3	4.46	0.30	3.85	0.54	−13.6	<0.05
SEsc (scaliness)	1	0.53	0.08	0.52	0.08	−1.9	n.s.
	2	0.56	0.06	0.64	0.02	13.4	<0.05
	3	0.62	0.02	0.56	0.09	−10.5	<0.05
SEsm (smoothness)	1	114.40	6.07	123.06	11.15	7.6	n.s.
	2	113.72	8.30	112.15	10.96	−1.4	n.s.
	3	151.74	11.49	127.54	7.67	−15.9	<0.05

**Table 4:** Components, manufacturers, and quantitative composition (% *w*/*w*) of the base cosmetic emulsion. This formulation served as the control (formulation 1) and as the vehicle for active ingredients in formulations 2 (with EGF loaded) and 3 (PRF loaded).

Components	Manufacturer	Content [% *w*/*w* ±0.01]
Creagel^®^ EZ 7 (polyacrylamide, hydrogenated polydecene, polyoxyethyl lauryl ether-auto-emulsifier/viscosity regulator)	Créations Couleurs	9.8
Alphaflow^®^ 20 (hydrogenated polydecane)—light emollient (hydrogenated polydecene) improving skin feel/spreadability	Créations Couleurs	16.7
Microcare^®^ SB (sodium benzoate, potassium sorbate) preservation system	Thor GmbH	0.2
Demineralized water	---	73.3

**Table 5:** Chromatographic analysis parameters.

Chromatographic column	Zorbax SB-C18 (250 mm × 4.6 mm; particle size 50 μm)
Eluent	Isocratic (Phase A: 80%; Phase B: 20%)
Phase composition	A: 7.5 mM sodium phosphate buffer (pH 6.8); B: acetonitrile
Moving phase flow velocity	0.6 mL/min
Injection volume	10 μL
Analysis time	25 min
Detector	UV-Vis
Analytical wavelength	214 nm

Good linearity (r ≥ 0.999) was achieved in the range of 0.5–100 μg/mL, and the LOD and LOQ values were 0.5 and 1.5 ng/mL, respectively. S/N ≈ 111, whereas the USP tailing factor (at 5% height) was equal to 1.15.

## Data Availability

The original contributions presented in this study are included in the article. Further inquiries can be directed to the corresponding authors.

## Abbreviations

The following abbreviations are used in this manuscript:

EGF	epidermal growth factor
PRF	platelet-rich fibrin
TEWL	trans-epidermal water loss
HPCL	high-performance liquid chromatography
